# How Much to Eat

**Published:** 1870-01

**Authors:** 


### HOW MUCH TO EAT.

All who employ workers should understand that it is a poor economy to stint them at the table. The man who eats the most can do the most work, for the simple reason that the power to work is obtained from the food we eat, and cannot be had from any other source. We met a New Yorker in Heidelberg last autumn, who had resided there for several years, and in speaking of the laboring classes, he said, that one Yankee would do as much work in one day as five Dutchmen. We subsequently found that there was truthfulness in the statement. It is absolutely painful to see a German laborer work. He moves so slowly. In the first place, he must have a pipe in his mouth; in the second place, if he is about to drive a spike in a rail, he looks around deliberately to see whether there is any possibility of knocking a hole in somebody's head; if he has to turn round, he raises his sleepy eyes to see if he will come in contact with somebody else, as the jar would discompose him, and a Dutchman don't want to be discomposed, it hurts his feelings, he don't like the idea of being excited; he would rather take a kick and do nothing; and then he rubs the kicked place so leisurely. The reason of his stolidity is — (minus) meat; + (plus) lager and pipe. The food of the lower classes of Germans is scant and poor; it is watery; so much soup, so much vegetable. A Dutchman makes two meals out of the rations for one. If you give a Yankee two pounds of meat for his dinner, he will eat it up and be done with it. Give a Dutchman two pounds of meat, and he would boil it to rags and shreds to-day, and feed a whole family on it; to-morrow he and his would feed on the shreds aforesaid. Hence it has been observed, that when a Dutchman comes to New York to work, it requires about one month's good eating before he is able to do as much work in a day as an American. There are two elements in the food we eat —warmth and power to work. These elements are in different proportions in each article; but it is found that in the same amount of various mixtures of food, these elements bear about the same proportion. Thus the benign Architect has built the house we live in—this material frame—under such regulations, that nature instinctively calls for the foods which have the proper proportion of the needed elements. If, for a few days, we take food which has more than the proper proportions, we are said to "get tired of it," we "crave" something else, which supplements the deficient material. A healthy laboring man, weighing a hundred and forty pounds, needs daily about one pound, or a little more, of the warming and power-producing elements called albuminate, to strengthen and repair, add carbon, or warmth, to keep alive. These keep him in health, and in working order. To obtain this pound of elements, he must eat about five pounds of meat and vegetables, the remaining four pounds being waste matter, and which being entirely useless to the body, must pass out of it daily, as we eat daily. If not, there is a clogging up of the system, which causes an infinite variety of symptoms, making life a misery. Hence the evil effects of costiveness, of a failure of the bowels to act every day; if there is such a failure, do not eat a particle except bread and water until they do act. This alone would prevent an infinite amount of sickness.

